# Supplementary figures and images for: Transcription of Clock Genes in Medulloblastoma
Source: Cancers (Basel). 2025 Feb 8;17(4):575. doi: 10.3390/cancers17040575 (PMC11852889; doi:10.3390/cancers17040575)

## Slide 1
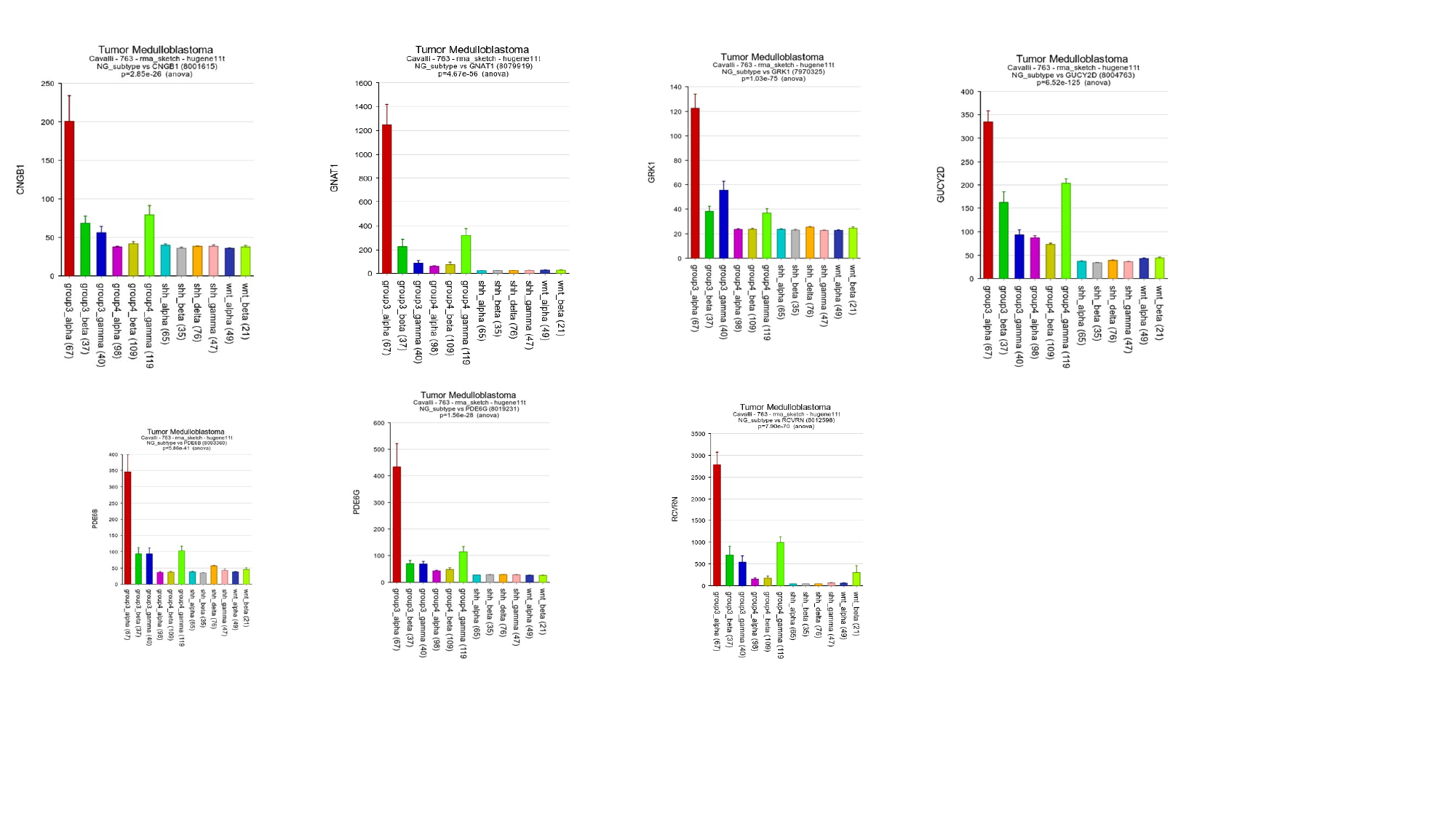

Supplement: Supplementary file 1 [file cancers-17-00575-s001.zip › cancers-3396971-supplementary.pptx]
